# Forest cutting and impacts on carbon in the eastern United States

**DOI:** 10.1038/srep03547

**Published:** 2013-12-19

**Authors:** Decheng Zhou, Shuguang Liu, Jennifer Oeding, Shuqing Zhao

**Affiliations:** 1 College of Urban and Environmental Sciences, and Key Laboratory for Earth Surface Processes of the Ministry of Education, Peking University, Beijing 100871, China; 2 U.S. Geological Survey (USGS) Earth Resources Observation and Science (EROS) Center, Sioux Falls, South Dakota, 57198; 3 Stinger Ghaffarian Technologies, Contractor to the USGS EROS Center, Sioux Falls, South Dakota, 57198. Work performed under USGS contract G10PC00044

## Abstract

Forest cutting is a major anthropogenic disturbance that affects forest carbon (C) storage and fluxes. Yet its characteristics and impacts on C cycling are poorly understood over large areas. Using recent annualized forest inventory data, we estimated cutting-related loss of live biomass in the eastern United States was 168 Tg C yr^−1^ from 2002 to 2010 (with C loss per unit forest area of 1.07 Mg ha^−1^ yr^−1^), which is equivalent to 70% of the total U.S. forest C sink or 11% of the national annual CO_2_ emissions from fossil-fuel combustion over the same period. We further revealed that specific cutting-related C loss varied with cutting intensities, forest types, stand ages, and geographic locations. Our results provide new insights to the characteristics of forest harvesting activities in the eastern United States and highlight the significance of partial cutting to regional and national carbon budgets.

Forest cutting, broadly defined here as human activities that remove trees for timber or for converting forestlands into other land uses, is a major anthropogenic disturbance that affects terrestrial carbon (C) storage and fluxes from local to global scales^1^,^2^,^3^,^4^,^5^. The United Nations Collaborative Programme on Reducing Emissions from Deforestation and Forest Degradation (REDD) in Developing Countries has been put in place to combat climate change, and it requires a periodic estimation of the national C dynamics in forest ecosystems, especially in the managed forests^6^, solidifying the significance of accounting for the impacts of forest cutting on the C cycle at a national scale.

Forests in the United States experience major management activities and disturbances that strongly affect its C sink strength^5^,^7^,^8^,^9^. The size and characteristics of C loss due to forest cutting activities remain highly uncertain in the country. Although the overall annual rate of U.S. forest harvest appears fairly stable since the 1980s^10^,^11^, the estimated C removals in the conterminous United States range from 92 to as much as 145 Tg C yr^−1^ in recent decades^2^,^12^,^13^,^14^,^15^,^16^,^17^. In addition, there is a critical data gap on the characterization of partial cutting in the United States^4^,^8^,^11^,^16^,^18^,^19^.

The newly available annualized repetitive plot measurements collected by the U.S. Forest Service (USFS) through the Forest Inventory and Analysis (FIA) Program provide an unprecedentedly high-quality, consistent, and systematic dataset for quantifying and analyzing the characteristics of forest cutting activities in the country^10^,^20^. Unlike estimates from ecosystem models and remote sensing techniques that usually mix all information together, the inventory-based estimates represent the full range of forest types, age classes, climate zones, and management regimes; therefore, the dataset can isolate different type of disturbances^9^,^21^. Here, we present bottom-up estimates of the average annual C loss from live biomass related to forest cutting in the eastern United States using the newly available database (version 5.1) (http://www.fia.fs.fed.us/tools-data/default.asp). The overall goal of this study was to examine the extent at which cutting currently affects the forest C cycle in the eastern United States and how this effect varies with cutting intensities, forest types, age classes, and geographical locations. The focus of this study was the eastern states (Figure 1) as the FIA dataset for the western states do not contain the revisited plots essential for estimating C loss in the present research.

## Results

### Carbon loss of live biomass by cutting intensities, forest types, and stand ages

There was 155 million ha of forests in the eastern United States averaged from 2002 to 2010. The total C loss of live biomass induced by forest cutting was estimated to be 168 Tg yr^−1^ for the period 2002–2010 in the eastern United States, with the C loss per unit forest area of 1.08 Mg ha^−1^ yr^−1^, including 104 (62%) in bole, 29 (17%) in belowground, 28 (16%) in top-limbs, and 8 (5%) Tg yr^−1^ in stump.

Forest cutting with different cutting intensities accounted for various proportions of the total C loss (Figure 2). Partial cutting, as indicated by the cutting intensity less than 90%, contributed 74% to the total C loss and the rest was attributed to clear harvesting. We examined the contribution of cutting to total C loss at an intensity gradient with 10% intervals. For intensities less than 50%, the contribution of cutting to total C loss increased, and for intensities between 50 and 90%, it fluctuated. The cutting-related C loss for different forests also varied with cutting intensities (Figure 2). For example, although partial cutting was the dominant cutting practice across all forest types, clear cutting contributed much more to the cutting-related C loss from biomass for softwood forests (36%) than for both hardwood (18%) and mixed forests (19%).

The amount of C loss that resulted from cutting differed by forest type and cutting intensity (Figure 3, Table 1). Hardwood forests covered 68% of all forest area but accounted for 48% of the total C loss. In contrast, softwood forests contributed 45% of total C loss with its 25% coverage. Mixed forests were responsible for the remaining 7% of both the total C loss and total forest area in the eastern United States (Table 1). Consequently, the C loss per unit softwood area was 1.96 Mg ha^−1^ yr^−1^, almost 2.5 and 2.0 times that found in hardwood forest (0.79 Mg ha^−1^ yr^−1^) and mixed forests (1.07 Mg ha^−1^ yr^−1^), respectively. Overall, hardwood forests accounted for 54% of all C loss induced by partial cutting in eastern U.S. forests. In contrast, most of the C loss caused by clear cutting was from softwood forests, with a dominant share of 63% (Figure 3). The share of C loss from clear cutting and partial cutting for mixed forests was 6% and 8%, respectively.

Figure 4a shows the frequency distributions of the C loss along cutting age for different forest types. Overall, the C loss increased first with the cutting ages followed by a decrease across all forest groups but peaked at various age ranges. Softwood, mixed forests, and hardwood were most frequently cut at an age of 20–30, 50–60, and 60–70 years, respectively. Correspondingly, the higher cutting-induced C loss density occurred at a much younger cutting age for softwood than for mixed forests and hardwood (Figure 4b). For example, the C loss density for softwood increased substantially from 0.10 to 3.04 Mg ha^−1^ yr^−1^ before age 30, followed by a gradual decrease to 0.66 Mg ha^−1^ yr^−1^ until age > 100. In contrast, the C loss density of mixed forests increased substantially from 0.09 to 1.37 Mg ha^−1^ yr^−1^ for the age range < 40, followed by a large fluctuation, and that of hardwood increased steadily from 0.20 Mg C ha^−1^ yr^−1^ at stand age < 10 years to a plateau around 0.96 Mg C ha^−1^ yr^−1^ for age > 70.

Partial cutting was the dominant cutting practice regardless of forest type and cutting age, and the amount of total C loss induced by partial cutting varied by forest type and the stand age when the cutting occurred (Figures 2 and 5). Figure 5 summarizes the contributions of partial cutting-induced C fluxes to C loss for different forest types and stand ages. Partial cutting contributed a relatively consistent proportion (around 83%) to the C loss for hardwood over different age ranges, the contribution of partial cutting-induced C loss for mixed forests generally decreased with the increasing stand ages, and the contribution for softwood fluctuated with one evident low point at stand ages of 30–50 years when a great amount of live biomass was lost in a relatively small softwood area caused primarily by clear cutting.

### Regional variations of the cutting-related carbon loss in live biomass

The amount of C loss due to forest cutting varied greatly with geographic locations, ranging from approximately zero in northwest Iowa to as much as 7.0 Tg C yr^−1^ in southeast Georgia (Figure 6b). The density of C loss indicated a large geographical heterogeneity as well. A loss density of more than 2.4 Mg C ha^−1^ yr^−1^ was found in southwest Alabama, southwest Arkansas, southeast Georgia, southwest and northwest Louisiana, and the Northern Coastal Plain where a large amount of forestland area exists, while a loss density of less than 0.1 Mg C ha^−1^ yr^−1^ occurred in North Dakota, eastern South Dakota, northwest Iowa, central Florida, western Nebraska, and the Upland Flats in Indiana where forest coverage was relatively low (Figures 6a and c). Overall, the southern portion of the eastern United States experienced substantially more intensive cutting activities than the northern regions, indicating by a substantially larger amount and higher density of C loss than in the northern regions (Figure 6, Table 1). For example, the South Central and Southeast regions accounted for 42 and 31% of the total C loss, respectively, and the C loss per unit forest area in the two regions were more than twice that in the northern part of the eastern United States (Table 1).

Partial cutting was the major cutting activity in all regions of the eastern United States. However, the share of total C loss in those regions varied by geographic location, with the largest share in Northeast and Northern Prairie States (92%), followed by North Lake States (79%), Southeast (70%), and South Central (65%). Hardwood cutting contributed over four-fifths of all C loss in the northern regions, whereas softwood cutting accounted for about three-fifths of the C loss in the southern regions (Table 1). Comparatively, the largest C loss density occurred in South Central, followed by Southeast, Northeast, North Lake States, and Northern Prairie States regardless of forest type (Table 1).

## Discussion

The most commonly used indicator of forest cutting in the United States is volume removed, which has been tracked by the USFS FIA in a relatively consistent manner for a long time period^22^. Therefore, we compared our estimate of the bole C loss with multiple studies based on FIA inventories. Since our estimate is for the eastern United States (e.g., 104 Tg C yr^−1^ for 2002–2010), the total removal of bole (e.g., 128 Tg C yr^−1^) was calculated based on the assumption that the removal in the western United States accounts for 19% of the total removal in the conterminous United States, proposed by Oswalt *et al.*^23^, which is comparable to the average of previously published estimates (115 ± 19 Tg C yr^−1^) (Table 2)^8^,^12^,^13^,^14^,^15^,^16^,^17^,^24^,^25^,^26^. These estimates might not be comparable in a strict sense as they represented estimates for different time periods (experienced various land use practices) using different inventories and calculation methods. The purpose of this comparison is to provide consistency and verification check on our calculation procedures. However, most of the previous estimates are based on periodic inventories and empirical models or process models; the results were highly dependent on the capability of the inventories and the models in tracking forests changes^11^,^16^,^27^. Apparently, the varying sampling designs and data collection methods of periodic inventories would introduce large uncertainties into detecting the nation's forest dynamics by comparing the successive inventories directly^28^. In addition, the accuracy of the model, if utilized, depended strongly on the model parameterization^16^. In contrast, we estimated the bole C loss in live biomass using the re-measured plots in annualized forest inventory data directly. The high consistency of the collected data ensured an unprecedentedly direct and integrated quantification of U.S. forest cutting and its impacts on C dynamics in this study^10^,^20^.

Top-limbs, stump, and belowground biomass of the removed trees together were estimated to account for 38% of the total C loss in this study. These sectors can exert substantial impacts on the C cycle since 1) the top-limbs of the removed trees are an important source of woody debris, and their post-treatments have a great impact on the C cycle^29^; and 2) the cutting-related loss of live biomass in stump and belowground roots would increase the down deadwood in the forest ecosystem^30^. Unfortunately, all of the components were usually ignored or simplified in the cutting-related C accounting^15^,^16^,^24^,^25^. Therefore, it is important to consider the C dynamics of the other sectors of trees induced by forest cutting disturbances besides the bole biomass.

Partial cutting, usually ignored in large-scale C accounting^4^,^8^,^21^, was found to be the dominant activity in the eastern United States (Figure 2), which was broadly in agreement with earlier estimates^22^,^23^. We further revealed that partial cutting was the major cutting practices regardless of forest type, stand age, and geographic location (Figures 3 and 5, Table 1). The C changes following partial cutting differ greatly from the well-known clear-cutting events^31^. For instance, most studies reported a decrease in the total ecosystem C stocks following the direct removal of live tree biomass via clear cutting^32^,^33^. On the contrary, partial cutting was documented to exert variable impacts on the total ecosystem C stocks^34^,^35^. Thus, our results highlight the critical role of partial cutting in regional and global C budgets.

The cutting activities occurred at different rates among forest types. Overall, softwood forests experienced more intensive cutting activities than hardwood and mixed forests (Table 1), mainly because of the high productivity of softwood that attracted large investments in practicing high-intensity forestry^22^. However, hardwood cutting accounted for a larger amount of total C loss relative to softwood harvesting, which was attributed mainly to the substantially large forest area (Table 1) and high merchantable biomass of timber on the landscape taken by hardwood^23^. That justifies a comparable amount of C loss to softwood (hardwood vs. softwood: 81 vs. 75 Tg C yr^−1^) even with a significantly lower C loss per unit forest area (0.79 vs.1.96 Mg C ha^−1^ yr^−1^).

Softwood was mostly cut at a much younger age than hardwood, and mixed forest was in between (Figure 4a). Interestingly, the C loss density decreased substantially after a dramatic increase for softwood, but it remained nearly stable after a gradual increase for hardwood along cutting ages (Figure 4b). This feature can be attributed to both natural and economic factors. First, the frequency distributions of the forestland area across various forest types (Figure 7a) is closely linked to the C loss distributions (Figure 4a) over age gradients (with the square correlation coefficients of 0.86, 0.69, and 0.53 for hardwood, softwood, and mixed forests, respectively), suggesting the pre-disturbance forest area is a major factor in determining cutting events. Second, the rapid growth of softwood ensures younger-age harvesting in softwood^22^, which can be seen by the differences of frequency distributions between C loss and forest area (Figures 4a, 7 a, and 7 b) over age gradients. For example, the frequencies of C loss in age 20–60 for softwood were greater than the frequencies of forest area over the same age ranges (i.e., the ratio in Figure 7b was more than 1). By contrast, the large and stable C loss density in hardwood over age 60 may be due mainly to the high and stable pre-disturbance live C density in old-age hardwood^37^, indicating by a relative larger frequency in C loss than in hardwood forest areas over age 60.

Cutting-related C loss showed a large geographical heterogeneity. In the northern portion of the eastern United States, the Northeast experienced the largest C loss, followed by Northern Lake States and Northern Prairie States (Table 1), which can be primarily explained by the availability of their pre-disturbance live biomass^15^ or forest area. The region with a large forest area was estimated to share a large live C loss (Table 1). The southern regions of the eastern United States, however, accounted for a substantially greater amount of C loss and had a higher C loss per unit forest area than the North (Table 1), although their pre-disturbance live C densities are less than those in the Northeast^15^. This can be mostly attributed to the fast growth conditions and large area allocated to forestry use in the South and forest management policies^11^,^22^. First, the southern portion of the eastern United States contributed 54% to the forest area in the eastern United States (Table 1), and over half of the area was allocated to forestry use^36^, which provides a strong foundation for forest cutting activities. Second, the high productivity and rapid growth conditions in the South mean a high-return investment and thus this region usually experienced high-intensity forestry^22^,^37^. Finally, public policy greatly affected the rate of forest cutting. For example, timber harvest on federal lands in the Northwest declined since the enactment of Northwest Forest Plan in 1993^38^; consequently, harvests increased on private lands that were largely distributed in the southern portion of the eastern United States^10^,^36^.

This study estimated that the total cutting-related loss of live biomass in the eastern United States was 168 Tg C yr^−1^ in 2002–2010, which was equivalent to 70% of the total U.S. forest C sink (240 Tg C yr^−1^)^5^ and 11% of the national annual CO_2_ emissions from fossil-fuel combustion over the same period^39^, emphasizing a great potential to mitigate climate change by forest management.

However, the C loss estimated in this study does not equate to the net cutting-related C emissions as some of the dead biomass is not returned immediately to the atmosphere but remains stored in a durable status such as in wood products^19^,^40^, which (if long-lived) can be considered a C sink^8^. In contrast, emissions associated with forest cutting from combustion, decomposition of debris, disturbed soil, the slow decay of leaves, wood, and roots, and harvested wood products are potentially large sources of C to the atmosphere^12^,^16^, and the source is likely to be strengthened by the reduced C accumulation rate due to the removal of leaf area (which is the physiological basis for tree productivity^41^). These uncertainties demonstrate the importance of a systematic quantification of the C fate in each forest sector following forest cutting.

## Methods

### Materials

The USFS FIA Program (http://fia.fs.fed.us/) provides forest inventory data for the United States. The FIA database for each individual state can be downloaded from the FIA DataMart (http://fia.fs.fed.us/tools-data) as Microsoft® Access® databases.

FIA protocols have changed from a periodic inventory to an annualized survey with one sample plot roughly per 2,428 ha (http://fia.fs.fed.us/library/database-documentation). In the periodic inventories, a wide variety of plot designs and regionally defined attributes were used by different states. Some data attributes may not be populated or certain data may have been collected or computed differently^28^. Therefore, it is difficult to characterize forest cutting directly by comparing periodic inventories because of changing sampling designs and data collection procedures^22^.

In contrast, annual inventories, initiated sometime after 1999, depending on the state, use a nationally standardized plot design and common data collection procedures. Some methodology and attribute definitions have also been changed to improve the inventory^28^. Notably, about one-fifth of plots were re-measured every year (5-year cycle) using the same method during the annualized survey, with one revisited plot per roughly 10,491 ha per year^20^. The greater consistency in collecting data during the annualized survey provides a strong foundation for estimating large-scale changes of the nation's forest^10^.

### Analyses

**Figure 1:**
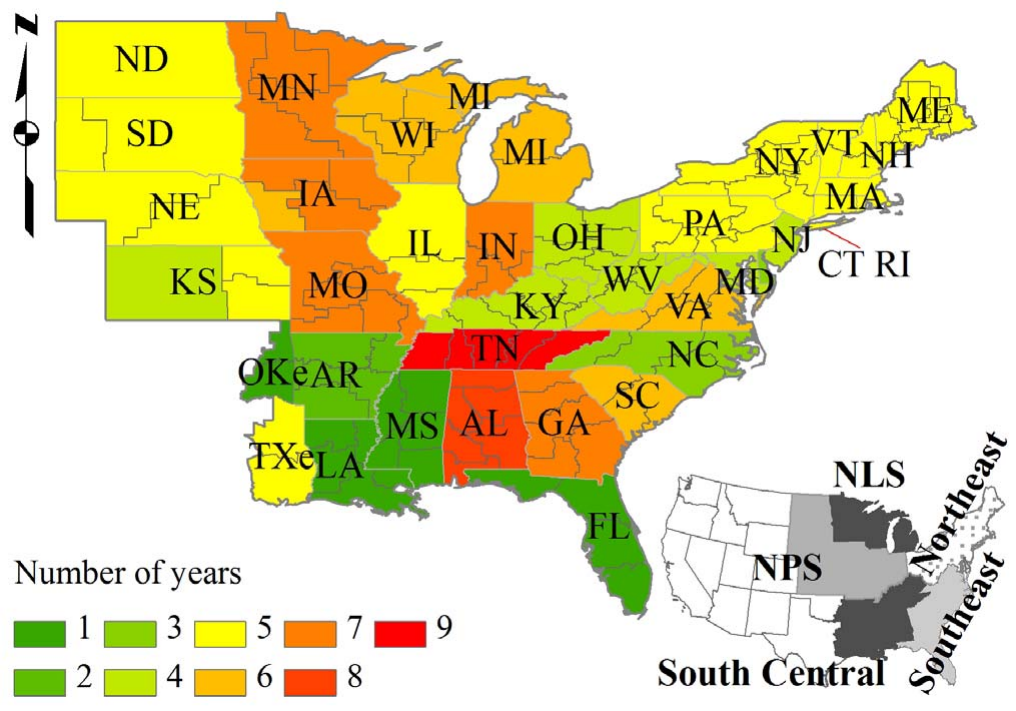
The locations of FIA survey units in the eastern United States with background color indicating the numbers of years from 2002 to 2010 that recorded the annualized data for tree removal. An FIA survey unit was defined as a group of counties in a state. NPS: Northern Prairie States; NLS: Northern Lake States; OKe: eastern Oklahoma; TXe: eastern Texas. Maps were generated using ArcGIS 9.3 (www.esri.com/software/arcgis).

**Figure 2:**
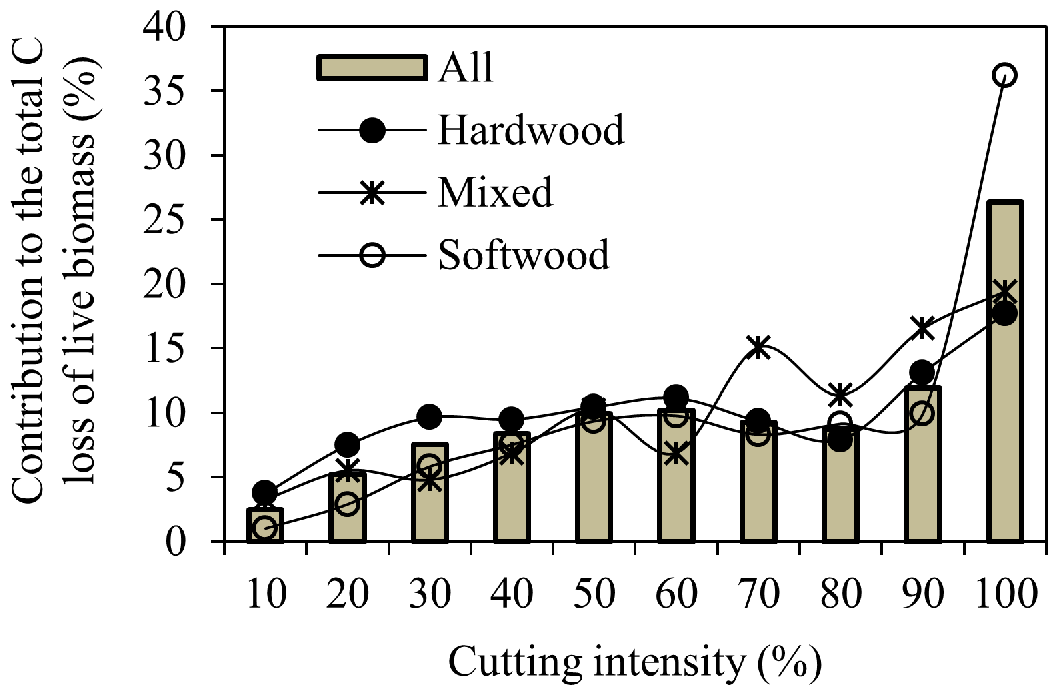
Contributions (%) of forest cutting with different cutting intensities to the total C loss of live biomass. Cutting intensities are defined as the percent of live biomass loss per sample plot caused by forest cutting during a revisiting cycle (around 5 years).

**Figure 3:**
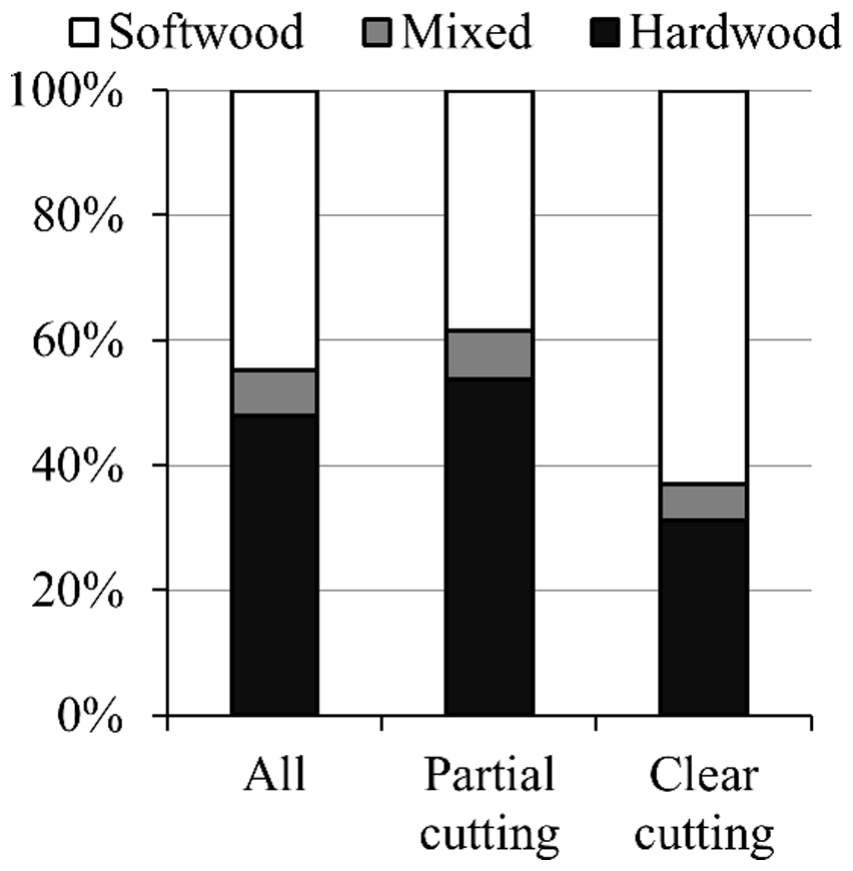
Contributions (%) of C removal caused by partial cutting, clear cutting, and all cutting events to the total C loss for different forests. Clear cutting referred to the cutting with an intensity of 90% or higher and the rest was defined as partial cutting.

**Figure 4:**
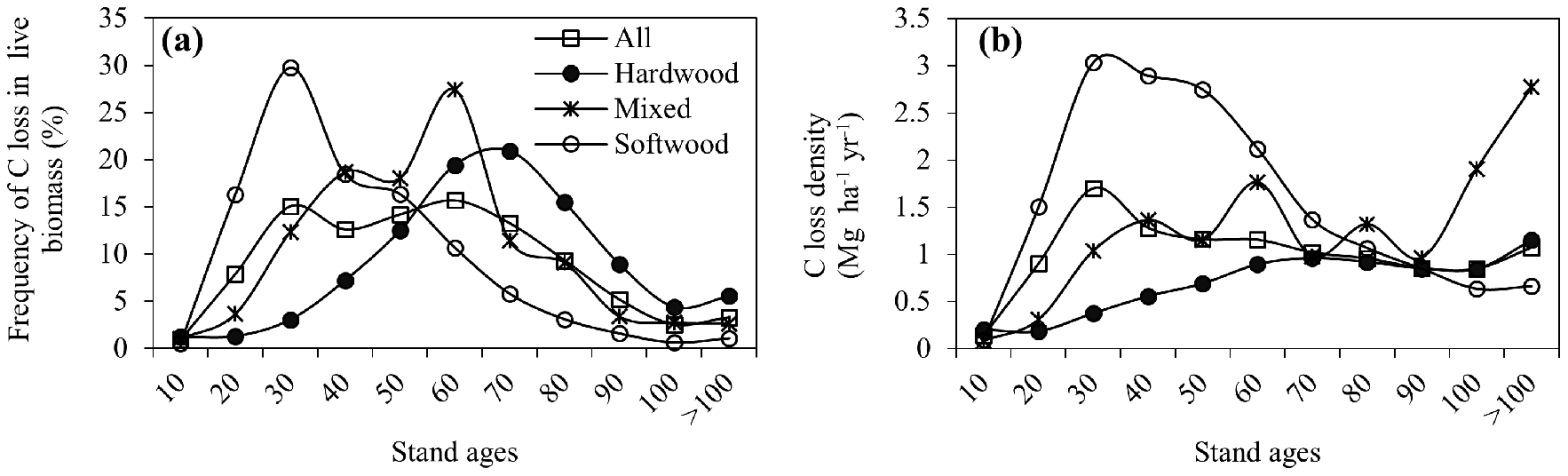
Frequency distributions (%) of total C loss in live biomass (a), and the C loss density (Mg C ha^−1^yr^−1^) (b) along cutting age gradients for different forest groups in the eastern United States.

**Figure 5:**
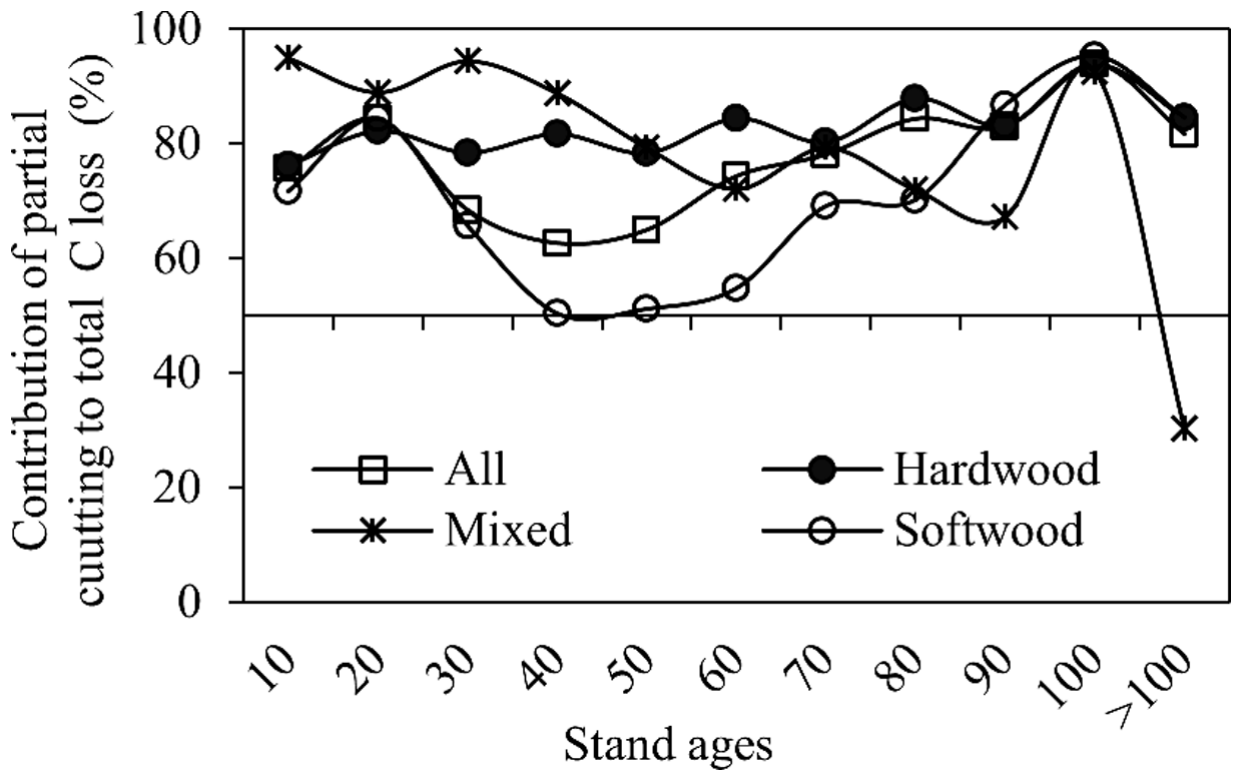
Contributions (%) of partial cutting-induced C fluxes to the total C loss of live biomass in different forests along cutting age sequences.

**Figure 6:**
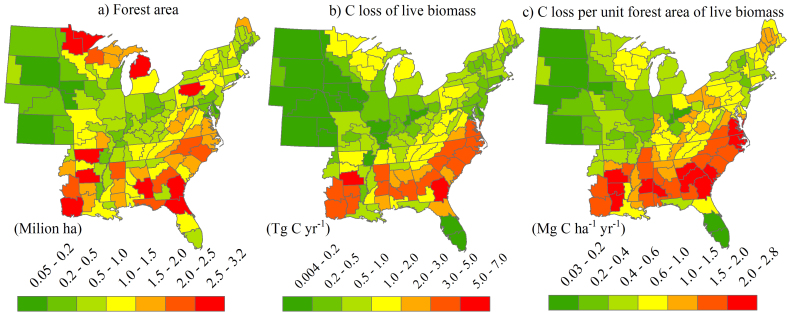
Spatial distribution of the forest area (a), the cutting-induced C loss in live biomass (b), and the C loss density (c) averaged over 2002–2010 for each FIA survey unit in the eastern United States. Maps were generated using ArcGIS 9.3 (www.esri.com/software/arcgis).

**Figure 7:**
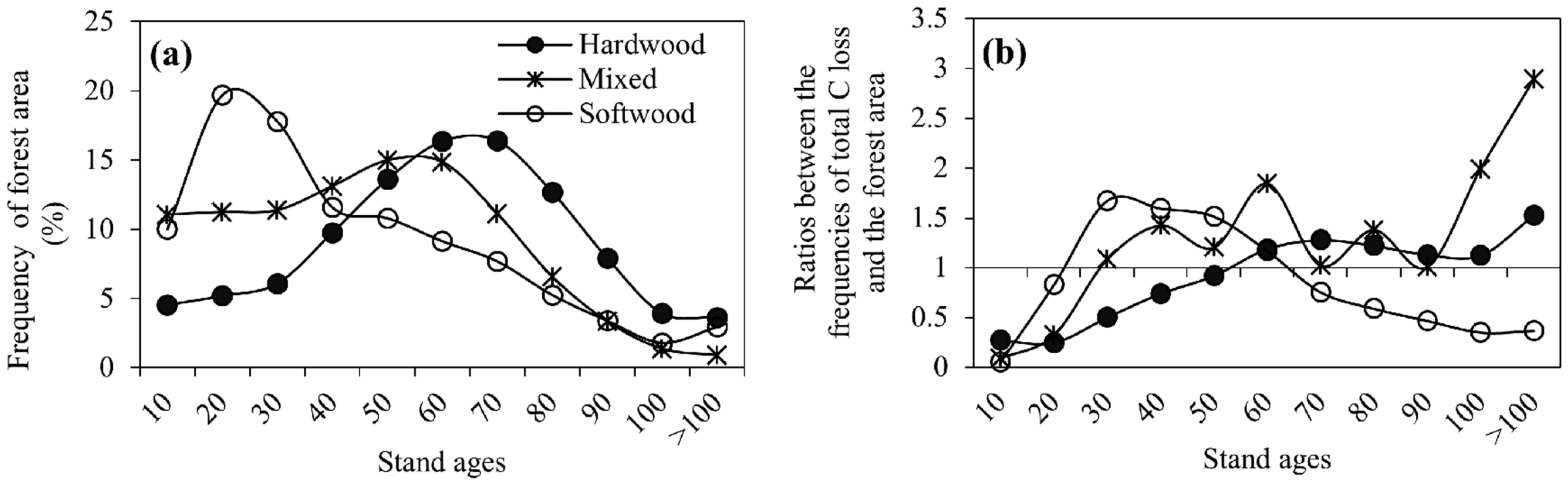
The frequency distributions of forest area (a), and the ratios between the frequencies of total C loss and the forest area derived from both Figures 4a and 7a (b) over different age ranges for each forest type.

FIA provides annual change data (derived from re-measured plots) in detail to tree level for change detection 1 to 6 years after the implementation of the annualized survey. We thus synthesized forest inventory information for the period that reported the annualized data (from 2002 or after to 2010) to estimate the mean annual cutting-related C loss of live biomass in the eastern United States at the FIA unit level (Figure 1). An FIA survey unit was defined as a group of counties in a state. We focused on 35 states and the eastern portions of Oklahoma and Texas (140 FIA units) in the eastern United States that together cover about half of the total U.S. forest area^27^. About 66% of the survey units recorded annual data in longer than 5 years (Figure 1). Trees harvested are reported in terms of sound cubic-foot volume, which is the annual removed volume of trees ≥ 5 inches in diameter of breast height (d.b.h.). The biomass loss of saplings (trees < 5 inches in d.b.h.) was not included in this study because their volume change was not available in the FIA dataset.

The live biomass (dry weight) loss of a tree induced by forest cutting was computed as the sum of the four parts of trees (i.e., bole, top-limbs, stump, and belowground roots)^42^: 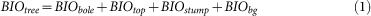
where *BIO_tree_* is the annual loss of live biomass (including bark but excluding foliage) for a tree that lived in the first survey and was removed in the second survey, and *BIO_bole_*, *BIO_top_*, *BIO_stump_*, and *BIO_bg_* are the biomass of the bole sector, top and limbs, wood and bark from ground level to 1 foot stump, and belowground roots, respectively.

The bole biomass calculation of each tree tallied on an FIA plot was specified and included wood and bark biomass. Each component (i.e., wood and bark) was calculated by multiplying its green volume (cuft) by the weight of 1 cubic-foot of water (62.4 lbs/cuft), which converts volume to weight, and then multiplying by the specific gravity of the component for the species: 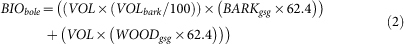
where *VOL* is the annual removal of sound cubic-foot wood volume of a tree ≥ 5 inches d.b.h. that has been tracked by the USFS FIA, *VOL_bark_* is bark volume as a percent of wood volume (unit: %), which is from Jenkins et al.^43^, and *BARK_gsg_* and *WOOD_gsg_* are the green specific gravity of bark and wood (green volume and oven-dry weight), respectively, which are from Miles and Smith^44^. Details on the *BIO_bole_* calculation are available in Heath *et al.*^42^.

*BIO_top_*, *BIO_stump_*, and *BIO_bg_* were estimated as follows: 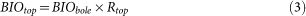

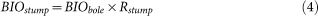

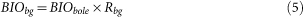
where *BIO_bole_* is biomass of bole, and *R_top_*, *R_stump_*, and *R_bg_* represent the average ratio of top-limbs, stump, and belowground biomass to *BIO_bole_*, respectively. These ratios were estimated by species and state from the USFS FIA for all live trees (not removed or dead). The cutting-induced C losses of trees were then summed to the total C loss at an FIA unit level as follows: 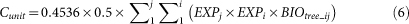
where *C_unit_* is the C loss per FIA unit, 0.4536 is the conversion factor of pounds to kilogram, 0.5 is the conversion coefficient of biomass to C^42^, *EXP_i_* and *EXP_j_* are the tree expansion factor and plot area expansion factor, which were used to scale each tree on a plot to a per-hectare basis and from plot level to FIA unit level, and *BIO_tree_ij_* represents the annual biomass loss of the *i* th tree in the *j* th plot caused by forest cutting. The C loss density was then obtained by subdividing the C loss by the corresponding forest area. The forest area was derived from the database directly using the method proposed by FIA^28^. Details on the expansion factors are available in Woudenberg *et al*.^28^

To fully understand the nature of forest cutting practices and their impacts on the C cycle, the cutting-related C losses were grouped by cutting intensities, forest types (hardwood, mixed, and softwood), stand ages, and geographic regions. Forest type and stand age were reported in the FIA dataset. The study area was divided into five regions^15^,^40^: Northern Prairie States, Northern Lake States, Northeast, Southeast, and South Central (Figure 1). Cutting intensity (CI) for each revisited plot was defined as the percentage of the total biomass loss that resulted from cutting: 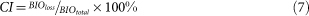
where *BIO_loss_* is the total cutting-induced C loss of live biomass during two consecutive periods, and *BIO_total_* is the total live biomass of the first of the two surveys. Clear cutting was defined as cutting with an intensity of 90% or higher, and the rest was referred to as partial cutting in this study.

## Figures and Tables

**Table 1:** Spatial patterns of forest cutting activities in the eastern United States

				Forest type									
	Total			Hardwood			Mixed			Softwood			Partial cutting
Region	a	b	ρC	c	d	ρC	c	d	ρC	c	d	ρC	e
Northern Prairie States	8.7	3.1	0.39	88.3	92.8	0.41	3.9	1.7	0.17	7.8	5.5	0.27	91.7
Northern Lake States	13.8	7.1	0.56	73.2	83.6	0.64	2.8	2.1	0.43	24.1	14.2	0.33	78.9
Northeast	24.2	16.9	0.76	82.1	84.3	0.76	3.4	3.0	0.66	14.6	12.7	0.64	92.3
Southeast	22.0	31.0	1.53	51.4	31.5	0.80	11.3	9.1	1.05	37.3	59.5	2.09	65.3
South Central	31.3	41.9	1.45	59.0	34.7	0.85	10.3	8.9	1.25	30.7	56.4	2.67	70.4
Total	100	100	1.07	67.6	48.0	0.79	7.2	7.1	1.10	25.2	44.9	1.96	74.0

a, Contribution of each region to the total forest area in the eastern United States (%).

b, Contribution of each region to the total C loss of live biomass in the eastern United States (%).

c, Contribution of the forest area of different forests to the total forest area in each region (%).

d, Contribution of the C loss in different forests to the total C loss of live biomass in each region (%).

e, Contribution of the C loss by partial cutting (with a cutting intensity less than 90%) to the total C loss of live biomass in each region (%).

ρC, C loss of live biomass per unit forest area (Mg C ha^−1^yr^−1^).

**Table 2:** Comparison of live C loss in bole (Tg C yr^−1^) for the conterminous United States from this study and a sample of previous estimates^a^

Source	Bole C removed	Periods
Hurtt et al., 2002^24^	92^C^	1980s
King et al., 2007^17^	145^C^	1980s
Pacala et al., 2001^8^	92^C^	1980s
Turner et al., 1995^12^	124	1980s
Birdsey & Heath, 1995^25^	126	1990
Heath & Smith, 2004^14^	105	1990s
Houghton, 1999^13^	92^C^	1990s
EPA, 2008^26^	132^C^	2005
Williams et al., 2012^16^	107	2005
Woodbury et al., 2007^15^	132^C^	2005
Average of previous studies	115 ± 19^d^	—
This study + western US	104 + 24^b^	2002–2010

^a^The bole C loss refers to the C of sound cubic-foot volume that is assumed to have been taken off site and entrained into wood products, equal to the C of volume removed used by the previous studies.

^b^We estimated the total C loss of bole in the conterminous United States by assuming the removals in the western United States accounted for 19% of the total removals in the conterminous United States as proposed by Oswalt *et al.*^23^.

^c^The estimates were derived from a synthesis by Williams *et al.*^16^.

^d^Mean estimate ± Standard error.
